# Insights into Correlation among Surface‐Structure‐Activity of Cobalt‐Derived Pre‐Catalyst for Oxygen Evolution Reaction

**DOI:** 10.1002/advs.201902830

**Published:** 2020-01-21

**Authors:** Ruchun Li, Bihua Hu, Tongwen Yu, Haixin Chen, Yi Wang, Shuqin Song

**Affiliations:** ^1^ The Key Lab of Low‐carbon Chemistry and Energy Conservation of Guangdong Province School of Materials Science and Engineering School of Chemical Engineering and Technology Sun Yat‐sen University Guangzhou 510275 China

**Keywords:** oxygen evolution reaction, pre‐catalysts, self‐reconstruction, surface functionalization, surface‐structure‐activity

## Abstract

Rational design of unique pre‐catalysts for highly active catalysts toward catalyzing the oxygen evolution reaction (OER) is a great challenge. Herein, a Co‐derived pre‐catalyst that allows gradual exposure of CoOOH that acts as the active center for OER catalysis is obtained by both phosphate ion surface functionalization and Mo inner doping. The obtained catalyst reveals an excellent OER activity with a low overpotential of 265 mV at a current density of 10 mA cm^−2^ and good durability in alkaline electrolyte, which is comparable to the majority of Co‐based OER catalysts. Specifically, the surface functionalization produces lots of Co‐PO_4_ species with oxygen vacancies which can trigger the surface self‐reconstruction of pre‐catalyst for a favorable OER reaction. Density functional theory calculations reveal that the Mo doping optimizes adsorption‐free energy of *OOH formation and thus accelerates intrinsic electrocatalytic activity. Expanding on these explorations, a series of transition metal oxide pre‐catalysts are obtained using this general design strategy. The work offers a fundamental understanding toward the correlation among surface‐structure‐activity for the pre‐catalyst design.

## Graphical abstract

Unravelling the intrinsic mechanism, especially what the “true” catalyst for the oxygen evolution reaction (OER) is, is still highly challenging. Herein, a Co‐based pre‐catalyst is designed that enables rational control over exposure of true active CoOOH centers by in situ self‐reconstruction to deliver excellent electrocatalytic performance, representing one of the state‐of‐the‐art OER catalysts.

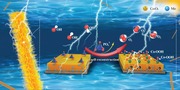

## 1 Introduction

Electrochemical water splitting holds great promise for large‐scale hydrogen production in the emerging clean energy economy.^1^, ^2^, ^3^, ^4^, ^5^, ^6^, ^7^, ^8^ At the anode side of water splitting, effective catalysts for the oxygen evolution reaction (OER) require a rational design to address the high overpotential challenge associated with the four‐electron transfer process to improve the overall water splitting efficiency.^9^, ^10^, ^11^, ^12^, ^13^, ^14^, ^15^ As state‐of‐the‐art OER catalysts, many efforts have been attempted to design particular structures, such as nanoneedle/nanowire,^15^, ^16^, ^17^ nanodendrites,^18^ mesoporous film,^19^ core–shell^20^, ^21^ with low content of precious Ir‐/Ru‐based oxides to address the cost and scarcity of these highly active metals which has severely restricted their widespread industrial deployment.^22^ To this end, non‐precious catalysts are an alternative and metals like cobalt, nickel, iron, and manganese are widely investigated.^1^, ^3^, ^23^, ^24^, ^25^, ^26^, ^27^, ^28^ Among those, cobalt derivatives including their oxides,^26^ hydroxides,^27^ phosphates,^4^ chalcogenides,^7^ borates,^28^ nitrides,^25^ and layer double hydroxides^23^ have been a main focus due to their excellent activity. Previous findings confirm that the real active center of the above catalysts is strongly associated with cobalt oxyhydroxide (CoOOH), which is usually formed at the surface of the pre‐catalyst under the oxidizing potential applied during the OER process.^1^, ^5^, ^9^, ^25^ Unfortunately, the commonly synthesized pure CoOOH phase is not the most highly active OER catalyst. Therefore, a fundamental insight to the pre‐catalysts design which would enable gradual exposure of more active CoOOH would greatly advance OER exploration with cobalt‐based catalysts.

Herein, we demonstrate, first, a rational design of the pre‐catalyst, P/Mo‐Co_3_O_4_@CC (phosphorous‐ and molybdenum‐doped cobalt oxide on carbon cloth) that can tune the CoOOH exposure process via in situ self‐reconstruction of Co‐PO_4_ and oxygen vacancies, and, secondly, how these effects can rationally promote OER activity. The P/Mo‐Co_3_O_4_@CC‐derived anode has a low overpotential of 265 mV at the current density of 10 mA cm^−2^ with good durability of more than 48 h in alkaline electrolyte. These results represent one of the most state‐of‐the‐art Co‐based OER catalysts. Furthermore, the appearance of CoOOH sites can be detected by in situ Raman spectra and advanced characterization. Together, these results show that the pre‐catalyst can be constructed using a rational strategy through surface functionalization with phosphate ions and Mo doping in Co_3_O_4_ on CC substrate. Density functional theory (DFT) calculations confirm that the Mo doping efficiently accelerates intrinsic electrocatalytic activity by optimizing adsorption free energy of *OOH formation and enhancing conductivity. By this general approach, we are able to systematically investigate a series of metal oxides that offers a fundamental understanding toward the correlation among surface‐structure‐activity and an effective strategy to design pre‐catalysts for high OER activity.

## 2 Results and Discussion

**Figure 1:**
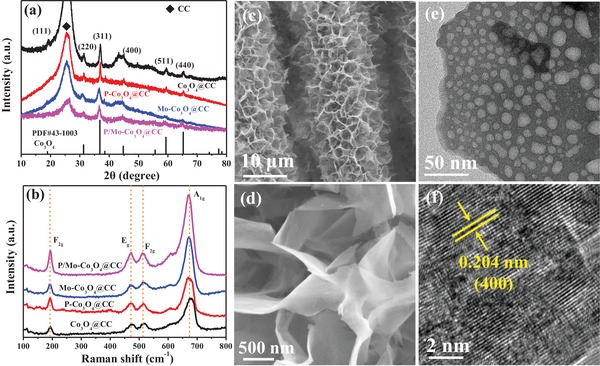
a) XRD patterns and b) Raman spectra of obtained samples; c,d) SEM and e,f) TEM images of P/Mo‐Co_3_O_4_.

The pre‐catalyst P/Mo‐Co_3_O_4_@CC was prepared as shown in Figure S1 in the Supporting Information. The Co‐based precursors were first modified onto the CC through hydrothermal treatment, and it was subsequently treated at high temperature in air atmosphere to obtain Mo‐doped Co_3_O_4_. Nanosheet arrays are found to be uniformly distributed onto the CC surface (Figure S2, Supporting Information), which could be attributed to the promotion to the nucleation process for the Co‐based oxides induced by the presence of Mo. The structure is highly stable and there is no change in morphology (Figures S3 and S4, Supporting Information) even after high‐temperature treatment. The prepared sample (Mo‐Co_3_O_4_) was further calcined in the presence of NaH_2_PO_2_ for phosphate ion surface functionalization to obtain the final pre‐catalyst P/Mo‐Co_3_O_4_@CC. The X‐ray diffraction (XRD) patterns are shown in **Figure** **1**a and Figure S5 in the Supporting Information. In Figure 1a, the strong peak located at ≈26.1° is ascribed to the substrate of CC. The other peaks at 19.2°, 31.5°, 36.9°, 44.8°, 59.5°, and 65.2° correspond to (111), (220), (311), (400), (511), and (440) crystal planes of Co_3_O_4_, respectively, according to PDF no. 43–1003 [cubic phase, space group: *Fd‐3m* (227)]. No obvious differences in XRD patterns of P/Mo‐Co_3_O_4_@CC are observed before and after doping, implying that no phase transformation occurs. It is consistent with the high‐resolution transmission electron microscopy (HRTEM) image (Figure 1f) that displays well lattice fringes with an interplanar distance of 0.204 nm in the P/Mo‐Co_3_O_4_ powders (scraped from P/Mo‐Co_3_O_4_@CC), corresponding to (400) plane of Co_3_O_4_. In addition, a typical Raman spectroscopy for spinel structure (AB_2_O_4_) is observed in Figure 1b. The Raman spectroscopy of P/Mo‐Co_3_O_4_@CC reveals typically Raman‐active modes including A1g (≈673 cm^−1^), Eg (≈472 cm^−1^), and F2g (≈194 and 515 cm^−1^) modes.^29^, ^30^, ^31^, ^32^ Furthermore, the morphology of 2D honeycomb‐like nanosheet arrays for P/Mo‐Co_3_O_4_@CC is observed (Figure 1c,d), and the Co, Mo, O, and P elements are uniformly distributed from the energy‐dispersive spectroscopy (EDS) elemental mapping result (Figure S6, Supporting Information). Surface reconstructions during functionalization are observed by TEM. This is evidenced from that the surface of P/Mo‐Co_3_O_4_ nanosheets has numerous disordered mesopores (Figure 1e). To further explore the pore structure, N_2_ adsorption–desorption isotherms was carried out. Type‐IV adsorption isotherms, which is a typical mesoporous structure behavior, are observed for P/Mo‐Co_3_O_4_. The Brunauer–Emmett–Teller (BET) surface area (inset of Figure S7a, Supporting Information) of Co_3_O_4_ and Mo‐Co_3_O_4_ is 46.1 and 73.8 m^2^ g^−1^, respectively, suggesting that Mo doping results in a larger BET surface area. This is due to the mesopores generation, which is confirmed by an obvious peak at 5.4 nm in pore size distribution curve for Mo‐Co_3_O_4_ (Figure S7b, Supporting Information) compared with that of Co_3_O_4_. It should be noted that the phosphate ion surface functionalization can increase the size of mesopore (Figure S7b, Supporting Information) as the BET surface area of the obtained P‐Co_3_O_4_ and P/Mo‐Co_3_O_4_ slightly decreases to 43.7 and 68.7 m^2^ g^−1^, respectively. This is beneficial to enhance the mass transport for OER.

**Figure 2:**
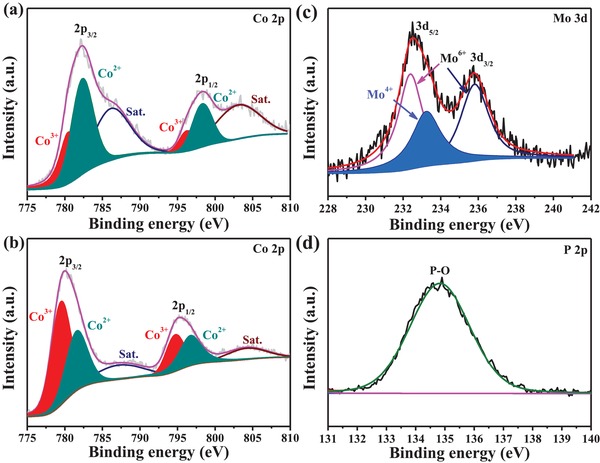
XPS analysis for the P/Mo‐Co_3_O_4_@CC and Co_3_O_4_@CC: a) Co 2p, c) Mo 3d, and d) P 2p of the P/Mo‐Co_3_O_4_@CC; b) Co 2p of the Co_3_O_4_@CC.

**Figure 3:**
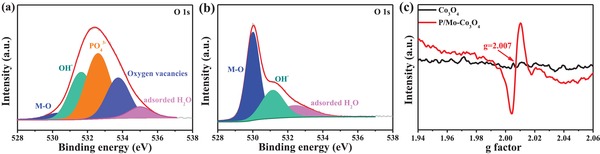
O 1s of the a) P/Mo‐Co_3_O_4_@CC and b) Co_3_O_4_@CC; and c) EPR spectra.

We further applied X‐ray photoelectron spectroscopy (XPS) to investigate the surface chemical composition and electronic structure. The presence of Co, Mo, O, and P (detailed in Table S1, Supporting Information) in P/Mo‐Co_3_O_4_@CC is confirmed in Figure S8 in the Supporting Information. The fitted Co 2p spectra of P/Mo‐Co_3_O_4_@CC (**Figure** **2**a) and Co_3_O_4_@CC (Figure 2b) relate to two different valence states of Co, including Co^3+^ and Co^2+^. The area ratio of Co^2+^/Co^3+^ for P/Mo‐Co_3_O_4_@CC is larger than that of Co_3_O_4_@CC (detailed in Table S2, Supporting Information), indicating that more Co^3+^ ions have been evolved into Co^2+^ ions in P/Mo‐Co_3_O_4_@CC after the surface functionalization. Generally, NaH_2_PO_2_ hydrate can be decomposed to generate PH_3_ gas under annealing treatment. In the presence of PH_3_ gas, as a reducing agent, Co_3_O_4_ can be reduced into Co_3_O_4−x_ during the annealing treatment, which accordingly leads to the reduction of valence state of Co.^33^ In the Mo 3d spectra (Figure 2c), two peaks located at ≈232.4 and ≈235.8 eV are assigned to Mo 3d5/2 and 3d3/2 of Mo^6+^ species, respectively. The peak at ≈233 eV is due to the presence of Mo^4+^.^34^ Moreover, the P 2p spectra (Figure 2d) reveal that the state of P species is almost in the form of PO_4_^3−^, which can be bonded with Co species to form Co‐PO_4_ species.^35^ The O 1s spectra of P/Mo‐Co_3_O_4_@CC (**Figure** **3**a) can be deconvoluted into five characteristic peaks at around 530.0, 531.5, 532.6, 533.8, and 535.1 eV, which are attributed to metal‐oxygen bond, hydroxyl groups (O_OH_), phosphate ion, oxygen vacancies (O_V_), and adsorbed water molecules, respectively.^33^, ^34^, ^35^, ^36^ While the O 1s spectra of Co_3_O_4_@CC in Figure 3b only exist metal–oxygen bond, hydroxyl groups, and adsorbed water molecules. For further exploring the existence of oxygen vacancies, the EPR spectroscopy was conducted. As shown in Figure 3c, the obtained P/Mo‐Co_3_O_4_ displays a strong signal at *g* ≈ 2.007, which is attributed to the oxygen vacancies, implying that abundant oxygen vacancies exist in P/Mo‐Co_3_O_4_. While, the corresponding signal is not observed in Co_3_O_4_, revealing no oxygen vacancies in Co_3_O_4_ crystal. These data evidence the formation of oxygen vacancies and Co‐PO_4_ species with a high Co^2+^/Co^3+^ ratio, expecting that the pre‐catalyst P/Mo‐Co_3_O_4_@CC will afford excellent electrocatalytic performance with the structural evolution undergoing OER.

**Figure 4:**
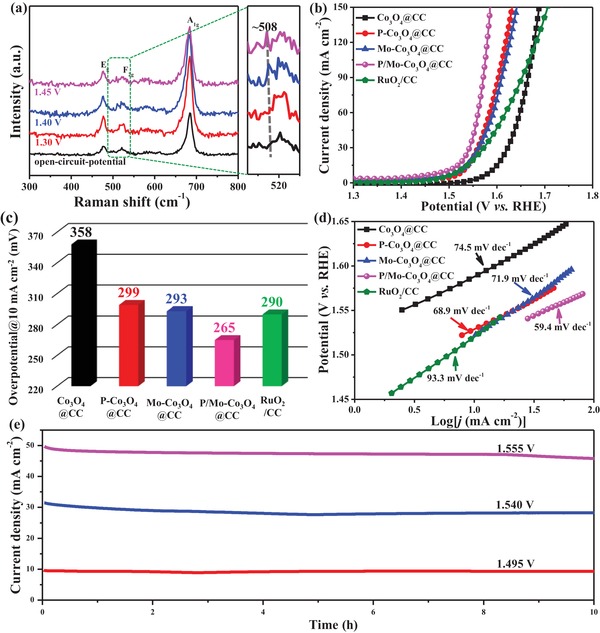
a) In situ Raman spectra of P/Mo‐Co_3_O_4_@CC at different potential in a 1 m KOH solution; b) the polarization curves at a scan rate of 5 mV s^−1^; c) the overpotential required for current density of 10 mA cm^−2^; d) Tafel plots; and e) the chronoamperometric curves for P/Mo‐Co_3_O_4_@CC at different potentials for 10 h.

In situ Raman measurements were employed to obtain mechanistic insight to the structure evolution process during OER. The peak at 508 cm^−1^, attributed to the formation of CoOOH, appears when the applied potential increases to 1.4 V [versus RHE (reversible hydrogen electrode), **Figure** **4**a]. No change for the peak is observed even when the potential is further increased to 1.45 V (near OER onset potential), indicating that CoOOH can be stabilized during the OER. These further confirm the formation of CoOOH on the pre‐catalyst surface, which can act as the true catalyst for OER.^5^, ^9^ More detailed characterization and theoretical calculations will be later discussed.

**Figure 5:**
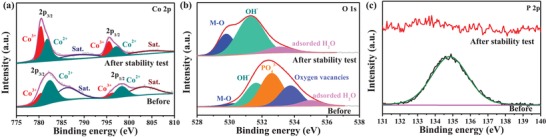
a) Co 2p, b) O 1s, and c) P 2p XPS spectra of P/Mo‐Co_3_O_4_@CC before and after stability test at 1.495 V for 10 h.

**Figure 6:**
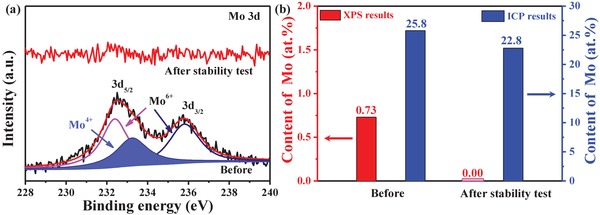
a) Mo 3d XPS spectra and b) the Mo content of P/Mo‐Co_3_O_4_@CC before and after stability test at 1.495 V for 10 h.

**Scheme 1:**
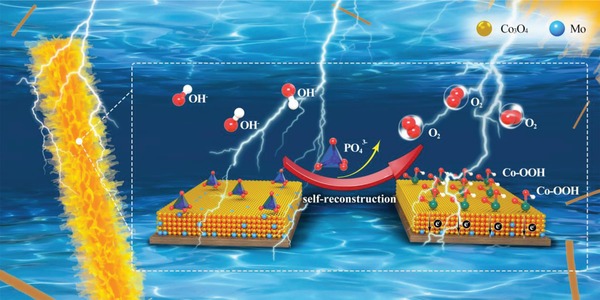
Schematic illustration of CoOOH exposure by in situ self‐reconstruction to catalyze OER.

The OER performance of the catalysts were further characterized in a standard three‐electrode set‐up in 1.0 m KOH solution. As shown in Figure S9 in the Supporting Information, the cyclic voltammogram (CV) curve of obtained material presents two redox couples, which correspond to the transformation of Co^2+^/Co^3+^ and to Co^3+^/Co^4+^.^37^ Thus, Co^4+^ species could probably serve as the crucial intermediate to enable formation of dioxygen for OER that is consistent with the previously reported results.^38^ The P/Mo‐Co_3_O_4_@CC shows an excellent OER performance with a small overpotential of 265 mV at 10 mA cm^−2^ (Figure 4b,c), which even outperforms the noble benchmark catalyst RuO_2_/CC (290 mV). The Tafel slope decreases dramatically from 93.3 mV dec^−1^ at RuO_2_/CC, 74.5 mV dec^−1^ at Co_3_O_4_@CC, 71.9 mV dec^−1^ at Mo‐Co_3_O_4_@CC, 68.9 mV dec^−1^ at P‐Co_3_O_4_@CC to 59.4 mV dec^−1^ at P/Mo‐Co_3_O_4_@CC (Figure 4d), suggesting the fastest OER kinetic reaction of the P/Mo‐Co_3_O_4_.^39^, ^40^, ^41^ The chronoamperometric curves at different potentials are shown in Figure 4e and Figure S10 in the Supporting Information. No obvious degradation is observed, suggesting its excellent stability for OER catalysis. In addition, a superior stability is found on the P/Mo‐Co_3_O_4_@CC as there are no noticeable morphology and crystal phase change observed in scanning electron microscopy (SEM) and XRD patterns (Figure S11, Supporting Information) after stability test for 10 h at 1.495 V. To gain insight into the chemical state and composition of highly active CoOOH generated at the surface, the pre‐catalyst after stability undergoing OER was particularly detected by XPS. As shown in Figure S12 in the Supporting Information, no changes are found for the C 1s, O 1s, and Co 2p. Specifically, the high‐resolution Co 2p spectra in **Figure** **5**a reveal that the Co^3+^ area is obviously increased because more CoOOH is formed during OER. This can be further confirmed by the high‐resolution O 1s spectra. The increased intensity of metal–oxygen bound and hydroxyl groups is accompanied by the disappearance of PO_4_^3−^ peak and oxygen vacancies (Figure 5b and Figure S13, Supporting Information), illustrating that the active Co‐PO_4_ species and oxygen vacancies have been reconstructed to form CoOOH for catalyzing OER. Thus, the existence of oxygen vacancies plays an important role in promoting the formation of active phase CoOOH for catalyzing OER. Simultaneously, the P 2p signal also disappears (Figure 5c and Table S3, Supporting Information) and the content of PO_4_^3−^ ions in electrolyte apparently increases (Figure S14, Supporting Information), further indicating that almost all of Co‐PO_4_ species have been converted into CoOOH at the high oxidation potential and PO_4_^3−^ ions can be finally dissolved into the electrolyte. This result is further verified by EDS investigation of P/Mo‐Co_3_O_4_@CC with different CV cycles from 0.8 to 1.7 V (versus RHE). As shown in Table S4 in the Supporting Information, the atomic content of P gradually decreases with an increasing of CV cycles, and it is dropped to zero after 60 CV cycles. These results indicate that PO_4_^3−^ can promote the CoOOH formation but does not participate in the OER process. In addition, some reported literatures also have turned out that the phosphates can act as a proton transport mediator at the catalyst surface.^42^ But, our results are not consistent with this conclusion. Surprisingly, the Mo 3d signal (**Figure** **6**a) cannot be observed by the XPS measurement after OER stability test, indicating the Mo element disappeared at the surface of P/Mo‐Co_3_O_4_@CC. A further investigation by EDS and inductively coupled plasma atomic emission spectroscopy (ICP‐AES, Figure 6b, and Figure S15 and Table S3, Supporting Information) suggests that the Mo element exists in the bulk material. Based on the above results, it can be concluded that the pre‐catalyst P/Mo‐Co_3_O_4_@CC can efficiently generate CoOOH via in situ self‐reconstruction of Co‐PO_4_ accompanying with oxygen vacancies, and then delivers an excellent OER activity in alkaline electrolyte, as shown in **Scheme** **1**. The true OER catalyst is core‐shell CoOOH@Mo‐Co_3_O_4_@CC (TEM image in Figure S16, Supporting Information).

These intriguing findings promote us to explore the intrinsic origin for the excellent performance of the catalyst. The effects of Mo doping and PO_4_^3−^ surface functionalization were further investigated. The Mo doping with different Mo/Co molar ratios (Figure S17, Supporting Information) can efficiently improve the OER performance compared with the poorly performed Co_3_O_4_@CC. The optimized Mo/Co ratio with 3/13 can efficiently decrease the overpotential required to reach 10 mA cm^−2^ from 358 mV at Co_3_O_4_@CC to 293 mV at Mo‐Co_3_O_4_@CC. For the PO_4_^3−^ surface functionalization, the electrochemical results are summarized in Figure S18 in the Supporting Information. Detailedly, a “volcano‐like” trend is observed in Figure S18b in the Supporting Information. The OER electrocatalytic activity is significantly enhanced when 500.0 mg P sources is used, reaching the lowest overpotential of 265 mV at 10 mA cm^−2^. Whereas, the OER activity decreases at the catalyst with lower P dosage of 200.0 mg or higher P dosage of 1000.0 mg. The electrochemical surface area (ECSA) of different electrodes was estimated to understand PO_4_^3−^ surface functionalization influence. The larger ECSA, the more exposed active sites, and it will be easier to form CoOOH for OER catalysis. Based on the CV results (Figure S19, Supporting Information), the double layer capacitance (*C*_dl_) values (Figure S20, Supporting Information) of Co_3_O_4_@CC, Mo‐Co_3_O_4_@CC, P‐Co_3_O_4_@CC, and P/Mo‐Co_3_O_4_@CC are 2.8, 3.5, 9.3, and 9.4 mF cm^−2^, respectively, and the corresponding ECSA values are calculated to be 70.0, 87.5, 232.5, and 235.0 cm^−2^, respectively. Among them, the true catalyst (P/Mo‐Co_3_O_4_@CC) has the highest ECSA, suggesting that moderate PO_4_^3−^ functionalization on the surface of P/Mo‐Co_3_O_4_@CC can expose more catalytic sites. Therefore, combined with the XPS and ECSA results, it can be concluded that the active Co‐PO_4_ species obtained by the PO_4_^3−^ surface functionalization play a crucial role in the formation of reconstructed CoOOH.

**Figure 7:**
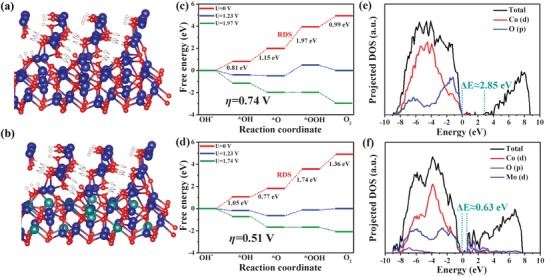
a,b) The structural diagrams, c,d) free‐energy diagrams and e,f) projected density of states of Co_3_O_4_ and P/Mo‐Co_3_O_4_ respectively. The blue, red, white, and olive spheres represent Co, O, H, and Mo atoms, respectively.

To gain more mechanistic insights, DFT calculation was conducted to simulate the OER process on true catalysts by establishing the corresponding theoretical models. Generally, in alkaline environment, the typical OER reaction proceeds according to the following mechanism:^43^, ^44^, ^45^, ^46^

(1) $${\text{OH}}^{-}+*\to *\text{OH}+{\text{e}}^{-}$$

(2) $$*\text{OH}+{\text{OH}}^{-}\to *\text{O}+{\text{H}}_{2}\text{O}+{\text{e}}^{-}$$

(3) $$*\text{O}+{\text{OH}}^{-}\to *\text{OOH}+{\text{e}}^{-}$$

(4) $$*\text{OOH}+{\text{OH}}^{-}\to *+{\text{O}}_{2}+{\text{H}}_{2}\text{O}+{\text{e}}^{-}$$

where * and *M represent the active site and the adsorbed intermediate on the active site, respectively. Distinguishing from many previous reports directly using original samples as models,^44^, ^45^, ^46^, ^47^ we take into account the fact that the active CoOOH catalytic phases will be formed on the surface of Co_3_O_4_ and P/Mo‐Co_3_O_4_ during OER reaction based on the above detailed exploration (Raman spectra and XPS spectra in Figures 4a, 5, and 6). Here, the optimized Co_3_O_4_ slab model was built by constructing a Co_3_O_4_@CoOOH heterostructure (**Figure** **7**a). Accordingly, the optimized P/Mo‐Co_3_O_4_ slab model was built by substituting some Co atoms by Mo atoms in Co_3_O_4_ of Co_3_O_4_@CoOOH heterostructure (Mo‐Co_3_O_4_@CoOOH, Figure 7b). The DFT results reveal that the rate determining step (RDS) of OER for Co_3_O_4_ and P/Mo‐Co_3_O_4_ is the formation of intermediates *OOH (step 3). The largest free energy change of the RDS in Co_3_O_4_ is 1.97 eV, corresponding to a 0.74 V theoretical onset overpotential (Figure 7c). After Mo doping, P/Mo‐Co_3_O_4_ possesses a lower free energy change (1.74 eV, Figure 7d) of the RDS than that of Co_3_O_4_, revealing a lower theoretical onset overpotential (0.51 V) and more favorable to OER kinetics. In addition, the density of state (DOS) of optimized models was also calculated to deeply elucidate inner Mo effect in the catalyst. As shown in Figure 7e, the bandgap between the valence and conduction bands of Co_3_O_4_@CoOOH model is around 2.85 eV. While, a smaller bandgap value (≈0.63 eV) is observed in Mo‐Co_3_O_4_@CoOOH (Figure 7f), implying a higher conductivity,^45^, ^47^ which is consistent with electrochemical impedance spectroscopy (EIS) results (Figure S21, Supporting Information). The total DFT results reveal that the inner Mo doping can promote OER kinetics and enhance the conductivity, and thus efficiently accelerating the intrinsic electrocatalytic activity.

The mechanism provides us an insight into the rational design of OER pre‐catalysts (Table S5, Supporting Information). We thus have prepared two similar catalysts P/Mo‐NiO@CC and P/Mo‐FeO_x_@CC with the same approach. The electrochemical results reveal a lower potential and smaller Tafel slope relative to that of NiO@CC and FeO_x_@CC (Figures S22 and S23, Supporting Information), indicating that the strategy of the inner doping by Mo and surface functionalization through PO_4_^3−^ could also be applicable to the other metal oxides, confirming a general strategy to prepare OER catalysts with high performance.

## 3 Conclusion

In summary, a Co‐based OER pre‐catalyst has been successfully developed that could continuously expose highly active sites due to the surface self‐reconstruction of Co‐PO_4_ species and oxygen vacancies under oxidizing potentials. And the structure of true catalyst was systematically investigated using in situ Raman and related advanced characterization. Furthermore, the surface functionalization with phosphate ions and oxygen vacancies can provide abundant active sites, meanwhile Mo doping efficiently boosts the intrinsic OER activity via the optimal adsorption‐free energy of *OOH by DFT calculation. The true catalyst realizes an excellent OER electrocatalytic activity and an attractive long‐term stability, and offers a dramatic understanding toward the correlation among surface‐structure‐activity for the pre‐catalyst design. In addition, our work also opens a general strategy to optimize metal oxides (CoO_x_, NiO, FeO_x_ etc.) for OER pre‐catalysts.

## 4 Experimental Section

*Materials*: Sodium molybdate dihydrate (Na_2_MoO_4_·2H_2_O, 99.0%), cobalt nitrate hexahydrate (Co(NO_3_)_2_·6H_2_O, 99%), sodium hypophosphite (NaH_2_PO_2_, 99.0%), and ammonium fluoride (NH_4_F, 98.0%) were purchased from Shanghai Aladdin, China. Ethanol and urea (CO(NH_2_)_2_, 99%) were obtained from Tianjin Chemical Reagent Co. China. CC was obtained from Taiwan CeTech. All chemical reagents were used as received without further purification.

*Synthesis of Co_3_O_4_@CC*: Typically, CC was successively cleaned by water, acetone and ethanol with ultrasonication for 30 min to remove impurities of surface. Then, the cleaned CC was calcined at 400 °C for 2 h under air to obtain hydrophilic surface. Thereafter, Co(NO_3_)_2_·6H_2_O (4.00 mmol), CO(NH_2_)_2_ (15.00 mmol), and NH_4_F (8.00 mmol) were dissolved in 50.0 mL of distilled water under continuously stirring for 20 min. The mixed solution and pre‐treated CC was transferred into a 100 mL‐Teflon‐lined stainless steel autoclave and heated up to 120 °C and kept at this temperature for 6 h. Then, the solution was naturally cooled down to room temperature and the as‐prepared precursors were washed with ethanol for three times and dried in a vacuum oven at 60 °C. Finally, Co_3_O_4_@CC catalyst was obtained by calcining the precursors at 400 °C in air for 3 h.

*Synthesis of Mo‐Co_3_O_4_@CC*: CoMo precursors on CC were synthesized by the same procedure except that Co(NO_3_)_2_·6H_2_O (3.25 mmol) and Na_2_MoO_4_·2H_2_O (0.75 mmol, Mo/Co = 3/13) were used as the metal sources. Finally, the resulting CoMo precursors were calcined at 400 °C in air for 3 h to obtain Mo‐Co_3_O_4_@CC. In addition, Mo doping was also obtained with 4.00 mmol total metal sources but different mole ratios of Mo/Co (1/15, 2/14, and 4/12).

*Synthesis of P/Mo‐Co_3_O_4_@CC and P‐Co_3_O_4_@CC*: The obtained Mo‐Co_3_O_4_@CC was put into the center of a tube furnace, and NaH_2_PO_2_ as P sources was placed at the upstream side of the furnace. Then, the furnace was heated up to 300 °C at a ramping rate of 10 °C min^−1^ and kept at the temperature under an N_2_ atmosphere for 3 h. The different dosage (200.0, 500.0, and 1000.0 mg) of P sources was investigated. The P‐Co_3_O_4_ also was prepared with 500.0 mg of P sources by the same method for comparison.

*Material Characterizations*: XRD measurements were performed by a D‐MAX 2200 VPC diffractometer with Cuα K radiation (40 kV, 26 mA). TEM images were taken on a JEM‐2010 (HR) at 120 and 300 kV. SEM was carried out by using a Quanta 400FEG. XPS tests were conducted by an ESCALAB 250. The obtained spectrograms were analyzed with Xpspeak 41 software. The nitrogen adsorption/desorption experiments were performed on Micromeritics ASAP2020. Before the measurements, the samples were dried at 90 °C for more than 18 h under vacuum conditions. The specific surface area was calculated by using the BET analysis. Raman spectra was investigated by the aid of a HORIBA Jobin Yvon, LabRam HR800 laser cofocal microspectrometry spectrograph. The ICP‐AES analysis was performed on a TJA IRIS(HR).

*Electrochemical Measurements*: The electrochemical measurements were carried out on an Auto84480 electrochemical station (Metrohm) in a conventional three‐electrode system. An Hg/HgO electrode (1.0 m KOH) and graphite paper (Qingdao Dongkai Graphite Co., Ltd.) were used as the reference and counter electrodes, respectively. Linear sweep voltammetric curves were recorded with a scan rate of 5 mV s^−1^. EIS was carried out from 100 kHz to 0.1 Hz with an amplitude of 5 mV. Current–time (*i*–*t*) responses were measured by using chronoamperometric measurements. The ECSA values were calculated based on the CVs with various scan rates at a potential range of 0.6–0.7 V (vs RHE). All the tested potentials were converted to versus RHE according to *E*_vs. RHE_ = *E*_vs. Hg/HgO_ + 0.098 V + 0.059 pH. All the polarization curves were shown with iR correction unless otherwise noted.

## Conflict of Interest

The authors declare no conflict of interest.

## Supporting information

Supporting Information
