# Supplementary material for: Insights into Correlation among Surface‐Structure‐Activity of Cobalt‐Derived Pre‐Catalyst for Oxygen Evolution Reaction
Source: Adv Sci (Weinh). 2020 Jan 21;7(5):1902830. doi: 10.1002/advs.201902830 (PMC7055576; doi:10.1002/advs.201902830)
Supplement: Supplementary file 1 — Supporting Information [file ADVS-7-1902830-s001.pdf]

Copyright WILEY-VCH Verlag GmbH & Co. KGaA, 69469 Weinheim, Germany, 2020.

ADVANCED SCIENCE
Open Access

Supporting Information

for *Adv. Sci.,* DOI: 10.1002/advs.201902830

Insights into Correlation among Surface-Structure-Activity of Cobalt-Derived Pre-Catalyst for Oxygen Evolution Reaction

Ruchun Li, Bihua Hu, Tongwen Yu, Haixin Chen, Yi Wang, and Shuqin Song**

Supporting Information

Insights into Correlation among Surface-Structure-Activity of Cobalt Derived Precatalyst for Oxygen Evolution Reaction

Ruchun Li, Bihua Hu, Tongwen Yu, Haixin Chen, Yi Wang, and Shuqin Song**

R. C. Li, B. H. Hu, Prof. T. W. Yu, H. X. Chen, Prof. Y. Wang, Prof. S. Q. Song
The Key Lab of Low-carbon Chemistry & Energy Conservation of Guangdong Province, School of Materials Science and Engineering, School of Chemical Engineering and Technology, Sun Yat-sen University, Guangzhou 510275, China
E-mail: wangyi76@mail.sysu.edu.cn (Y. Wang); stsssq@mail.sysu.edu.cn (S. Song)

1. The electrochemical surface area (ECSA):

The double layer capacitance (C_{dl}) was measured in the non-Faradaic potential range to estimate the electrochemical surface area (ECSA) of different electrodes according to equation:

$$ECSA=C_{dl}/C_s \qquad (S1)$$

where C_s is the general specific capacitance with 0.040 mF cm^{-2} in 1 M KOH solution.

2. DFT calculation

DFT calculations were conducted by using Vienna ab initio Simulation Package (VASP) with the generalized gradient approximation (GGA) parameterized by Perdew, Burke and Ernzerhof (PBE) for the exchange correlation functional. Energy cut off of 400 eV and applicable k-points of 4*2*1 were used. Besides, systemic energy tolerance of $1*10^{-5}$ and remaining total force of $1*10^{-3}$ were also employed. Co_3O_4 (110) surface was used to build the slab and each slab consisted of five layers with 2 * 1 supercell. To model the CoOOH surface, CoOOH (012) represented as 4-layer slabs was employed according to the reported literatures.[1,2] The CoOOH@Co_3O_4 and CoOOH@Mo-Co_3O_4 heterostructures were simulated as CoOOH adsorbed on top of 4 layers of a Co_3O_4 (110) surface. In this structure, the free energy (ΔG_{ads}) is obtained by

$$\Delta G_{ads} = \Delta E_{ads} + \Delta ZPE - T\Delta S \qquad (S2)$$

where ΔZPE and ΔS are the contributions to the free energy from the zero-point vibration energy and entropy, respectively.

$$\Delta E_{OH} = E(OH*) - E(*) - [E(H_2O - 1/2\ E(H_2))] \qquad (S3)$$

$$\Delta E_{O} = E(O*) - E(*) - [E(H_2O) - E(H_2)] \qquad (S4)$$

$$\Delta E_{OOH} = E(OOH*) - E(*) - [2E(H_2O) -3/2\ E(H_2)] \qquad (S5)$$

The Gibbs free energy change can be expressed as

$$\Delta G_1 = \Delta G_{OH} - eU \qquad (S6)$$

$$\Delta G_2 = \Delta G_{O} - \Delta G_{OH} - eU \qquad (S7)$$

$$\Delta G_3 = \Delta G_{OOH} - \Delta G_{O} - eU \qquad (S8)$$

$$\Delta G_4 = 4.92[eV] - \Delta G_{OOH} - eU \qquad (S9)$$

where U is the potential measured against reversible hydrogen electrode (RHE) at standard conditions.

The theoretical overpotential is then readily defined as:

$$\eta_{OER} = \max[\Delta G_1, \Delta G_2, \Delta G_3, \Delta G_4] - 1.23\ [eV] \qquad (S10)$$

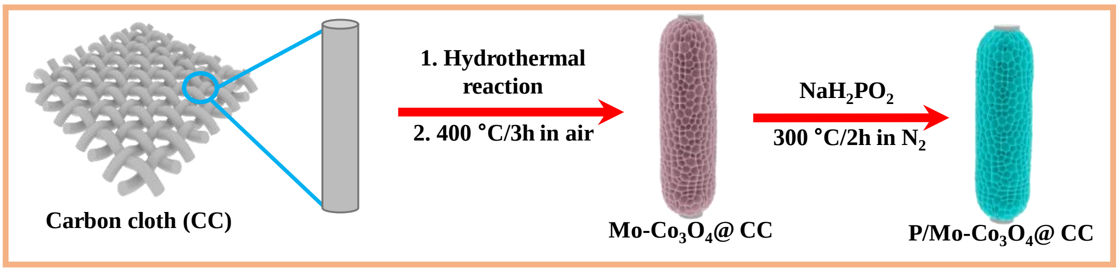

Figure S1. The schematic illustration for the preparation of pre-catalysts.

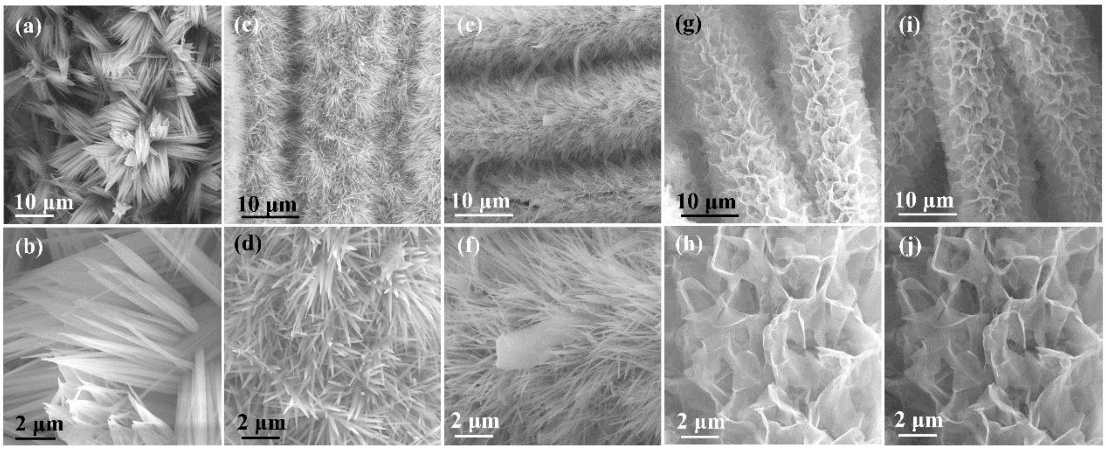

Figure S2. SEM images of Mo doped Co based precursors prepared by the different molar ratio of metal sources: (a-b) Mo/Co=0/16; (c-d) Mo/Co=1/15; (e-f) Mo/Co=2/14; (g-h) Mo/Co=3/13; (i-j) Mo/Co =4/12.

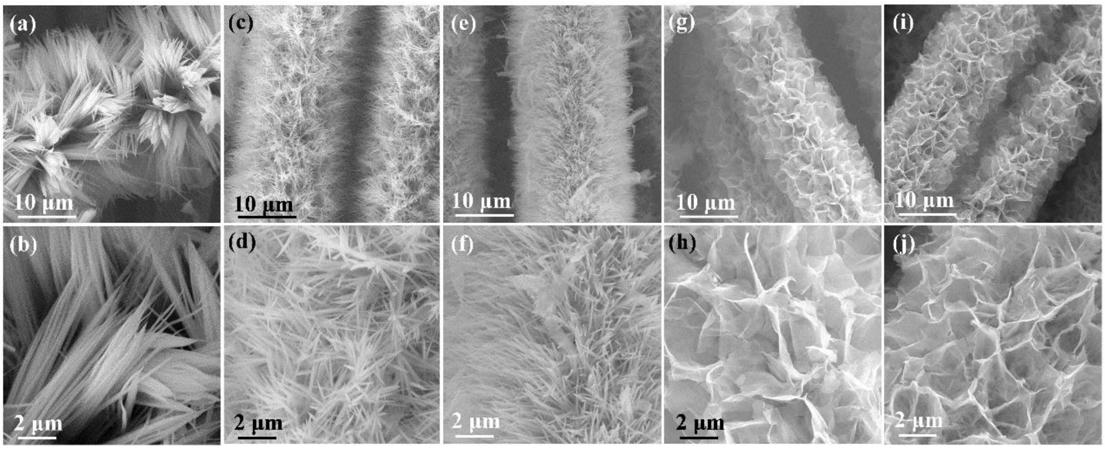

Figure S3. SEM images of different Mo doped Co_3O_4@CC prepared by the different molar ratio of metal sources: (a-b) Mo/Co=0/16; (c-d) Mo/Co=1/15; (e-f) Mo/Co=2/14; (g-h) Mo/Co=3/13; (i-j) Mo/Co =4/12.

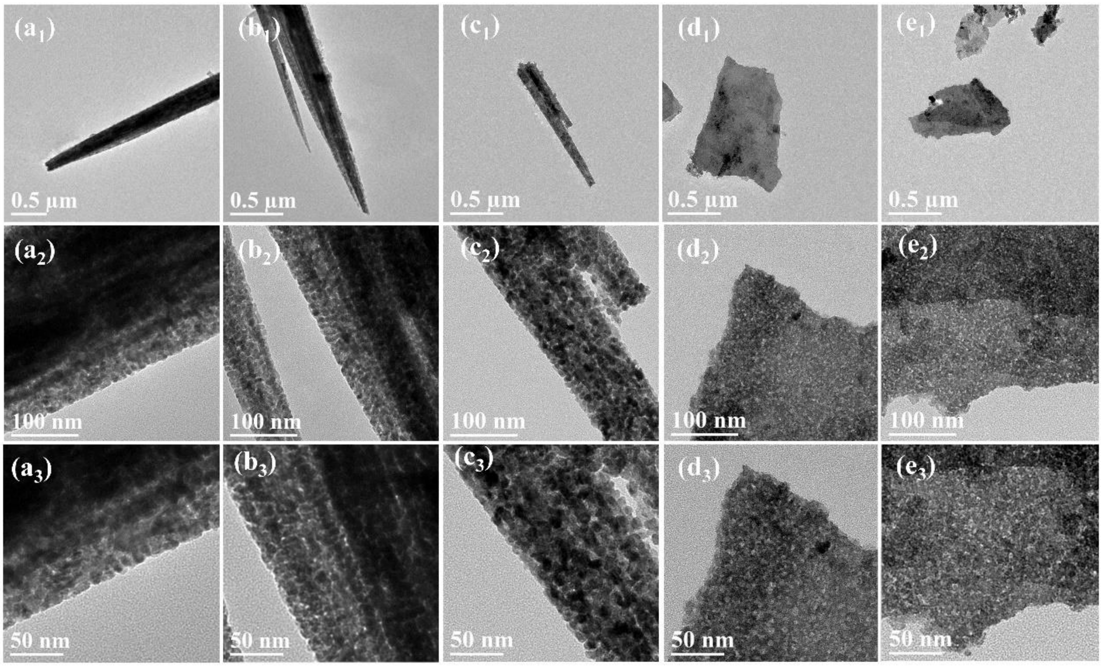

Figure S4. TEM images of different Mo doped Co_3O_4@CC prepared by the different molar ratio of metal sources: (a) Mo/Co=0/16; (b) Mo/Co =1/15; (c) Mo/Co =2/14; (d) Mo/Co =3/13; (e) Mo/Co =4/12.

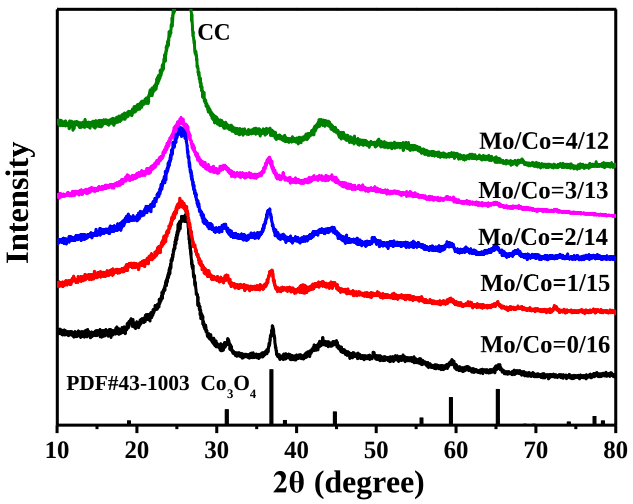

Figure S5. XRD patterns of different Mo doped Co_3O_4@CC prepared by the different molar ratio of metal sources.

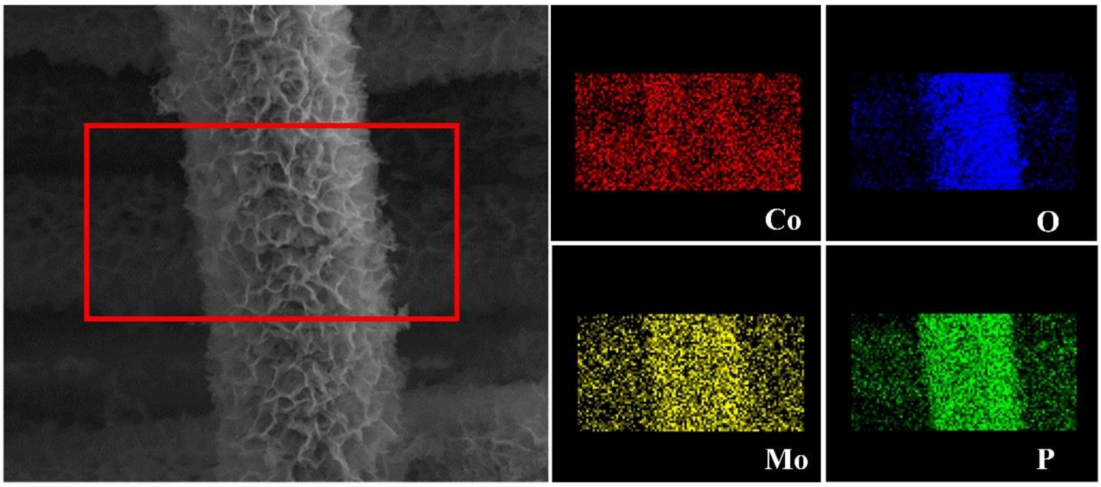

Figure S6. The elements mapping of P/Mo-Co_3O_4@CC.

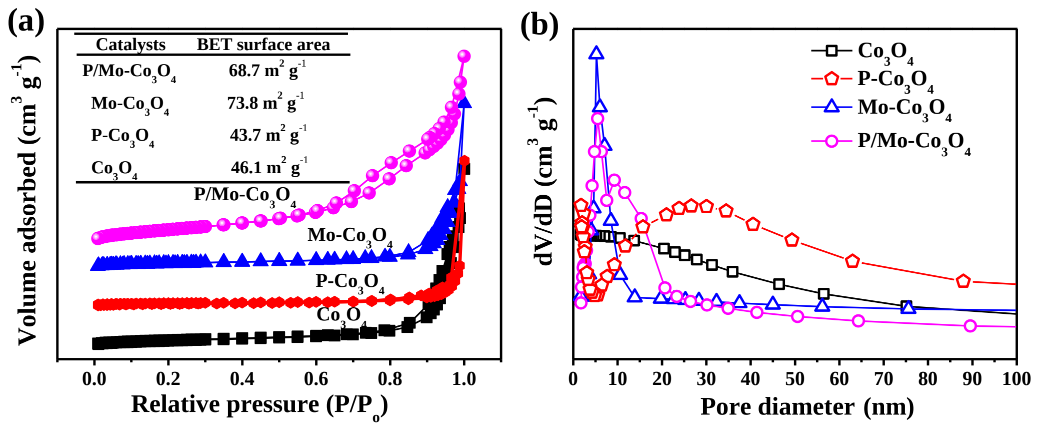

Figure S7. Nitrogen adsorption-desorption isotherms (a) and the corresponding pore size distributions (b) of P/Mo-Co_3O_4, Mo-Co_3O_4, P-Co_3O_4 and Co_3O_4.

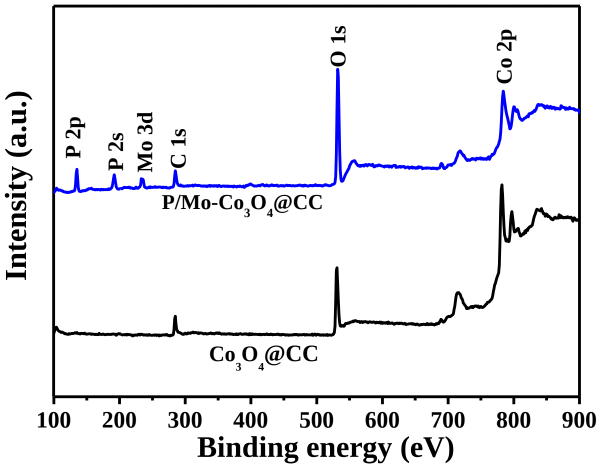

Figure S8. XPS spectra of obtained samples.

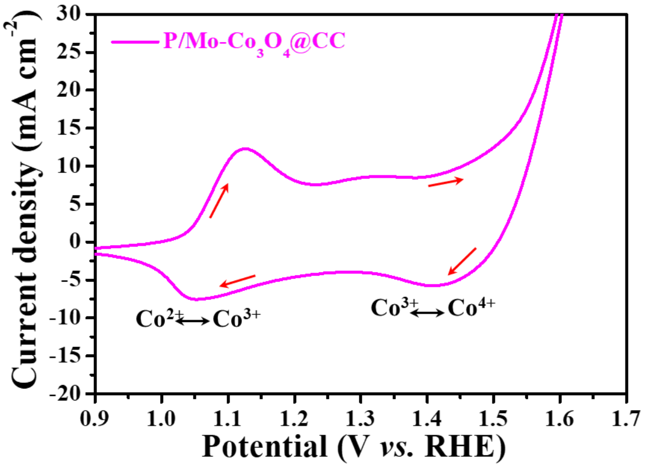

Figure S9. The CV curve of obtained P/Mo-Co_3O_4@CC.

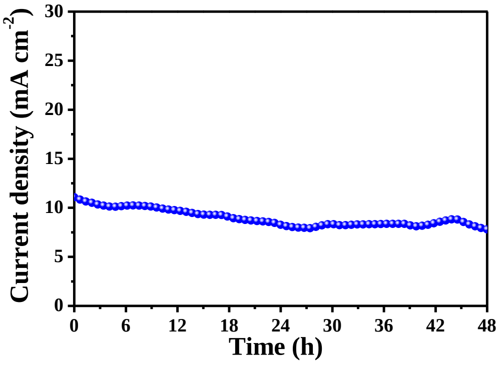

Figure S10. The stability test at 1.5 V for 48 h. To further evaluate the good long-term stability, the chronoamperometry experiment was carried out at 1.5 V for 48 h in Figure S10. The catalytic activity retention is as high as 80.1% after 48 h, indicating high stability of P/Mo-Co_3O_4@CC.

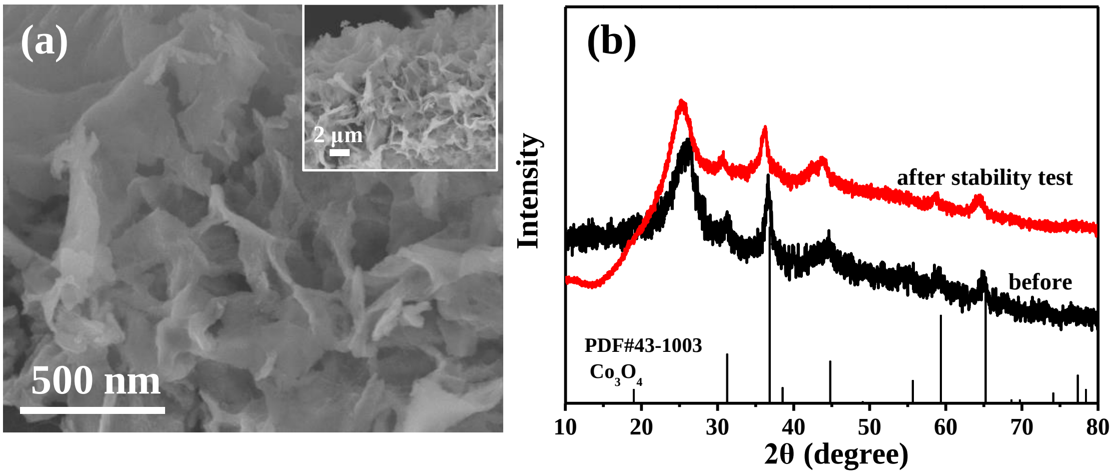

Figure S11. SEM images (a) and XRD patterns (b) of P/Mo-Co_3O_4@CC before and after stability test.

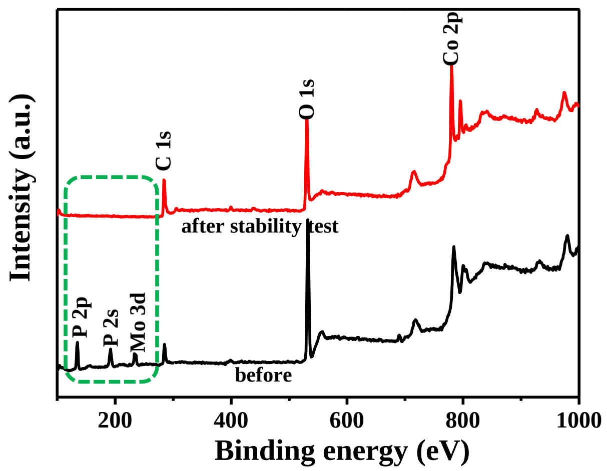

Figure S12. XPS spectra of P/Mo-Co_3O_4@CC before and after stability test.

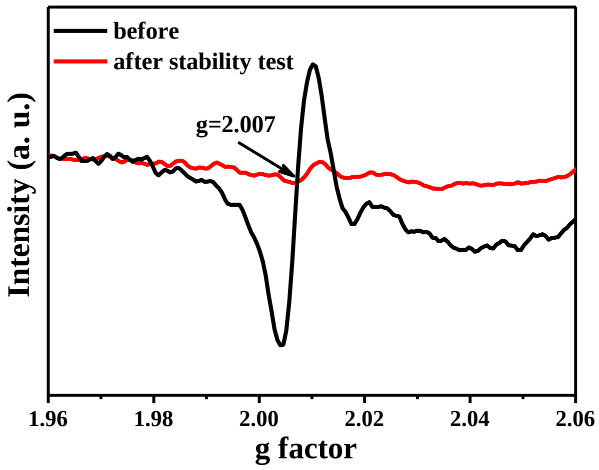

Figure S13. The EPR spectra before and after stability test.

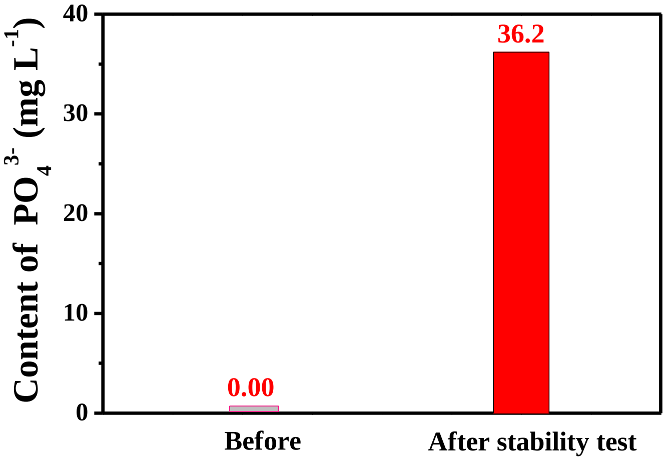

Figure S14. The content of PO_4^{3-} in measured electrolyte before and after stability test.

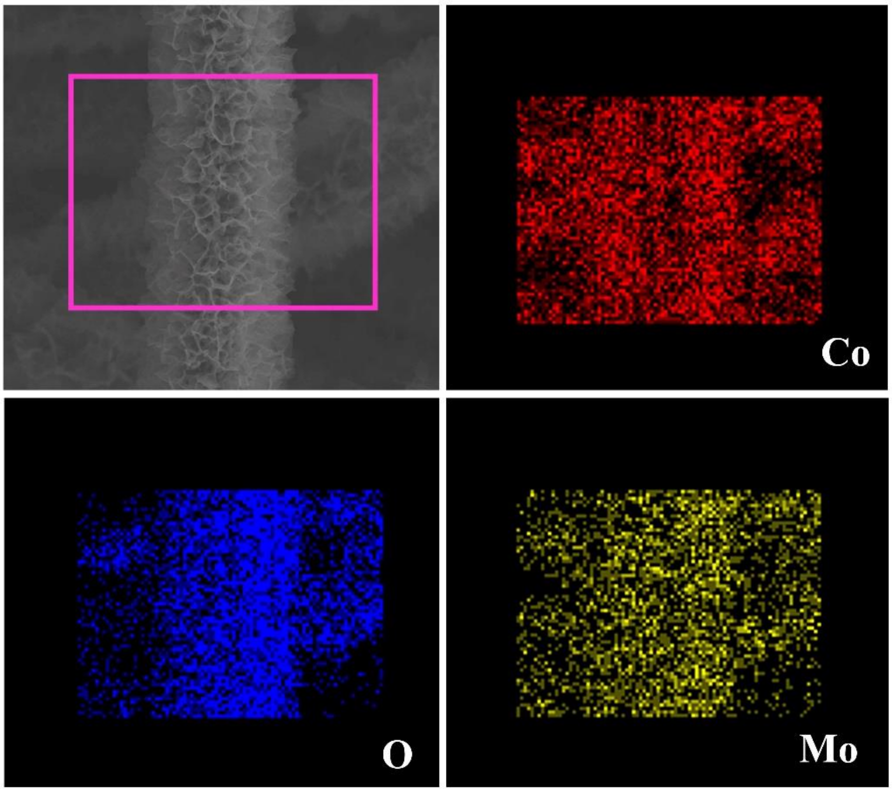

Figure S15. The elements mapping of P/Mo-Co_3O_4@CC after stability test.

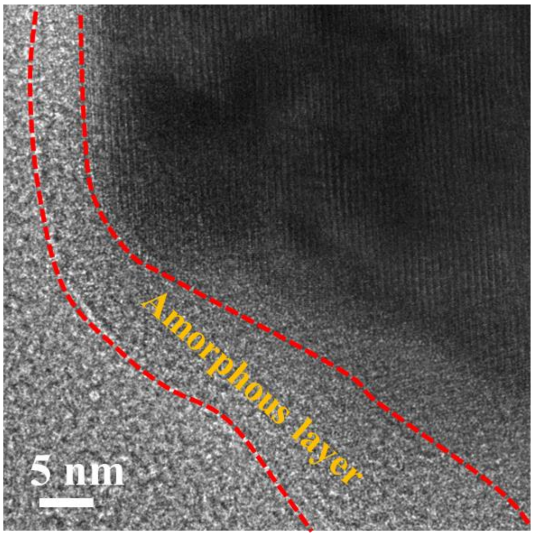

Figure S16. The formed amorphous CoOOH layer on precatalyst surface after stability test.

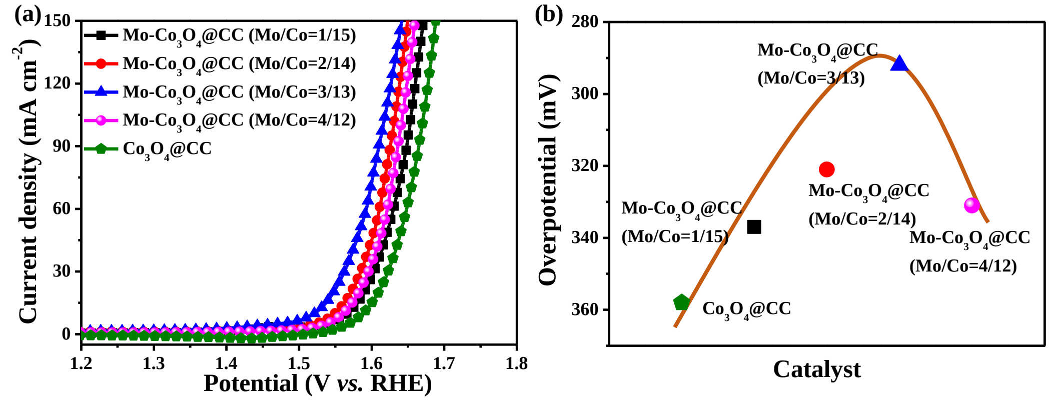

Figure S17. LSV curves of different Mo doped Co_3O_4@CC (a); the overpotential required for current density of 10 mA cm^{-2} (b). In order to optimize the Mo content, the OER activity depended on different Mo content in Co_3O_4 was investigated. The Mo-Co_3O_4@CC with the elemental ratio of Mo/Co=3/13 offers more excellent OER performance in the series samples, suggesting a rational optimization.

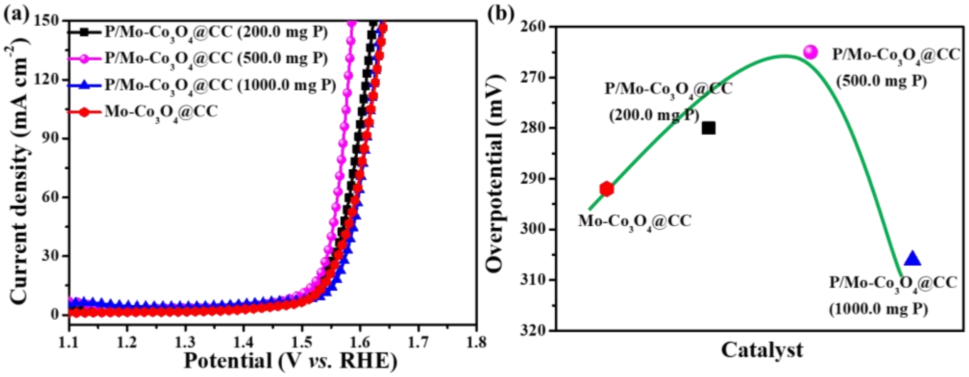

Figure S18. LSV curves of different P doped Mo-Co_3O_4@CC (a); the overpotential required for current density of 10 mA cm^{-2} (b).

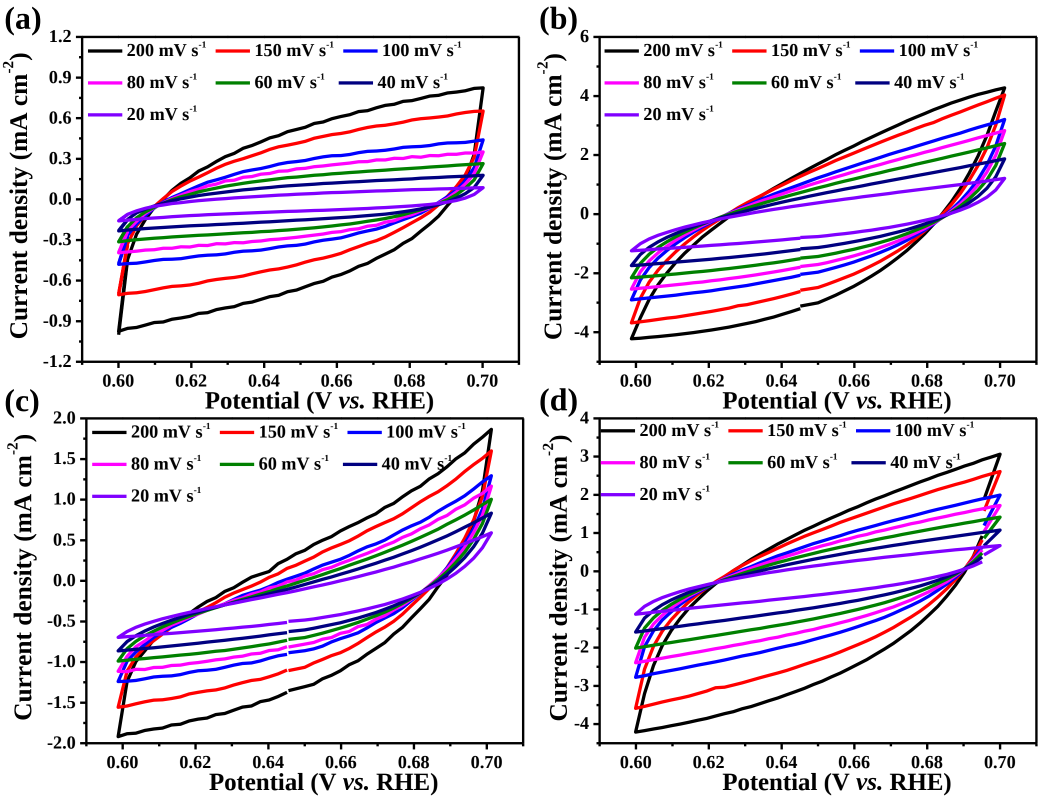

Figure S19. The CV curves of Co_3O_4@CC (a), P-Co_3O_4@CC (b), Mo-Co_3O_4@CC (c) and P/Mo-Co_3O_4@CC (d).

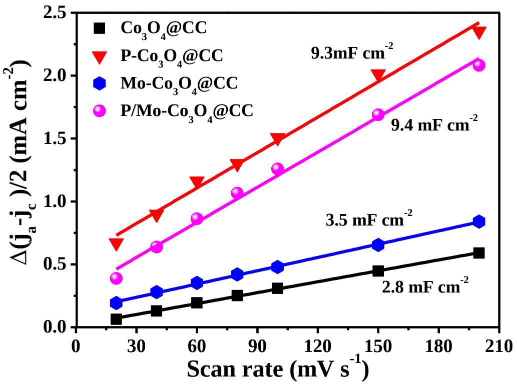

Figure S20. The capacitive currents at 0.65 V (*vs*. RHE) as a function of scan rates.

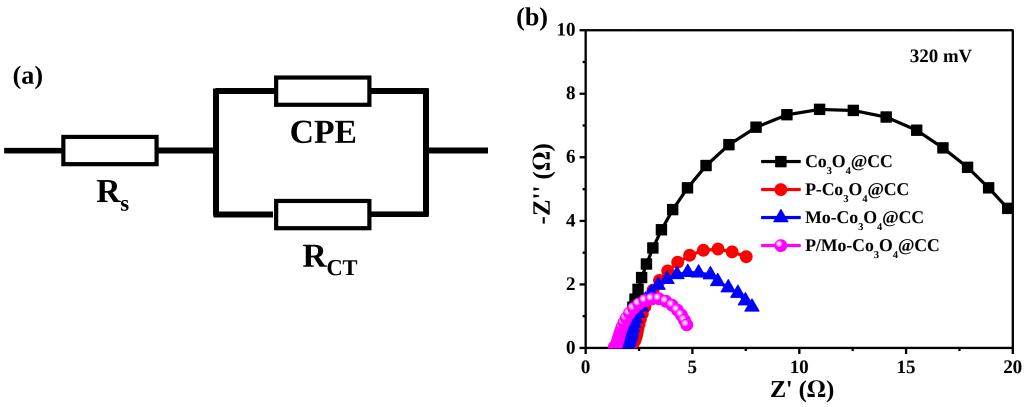

Figure S21. The equivalent circuit model (a) (Rs: electrolyte resistance, CPE: double layer capacity, Rct: charge transfer resistance); Nyquist plots (b).

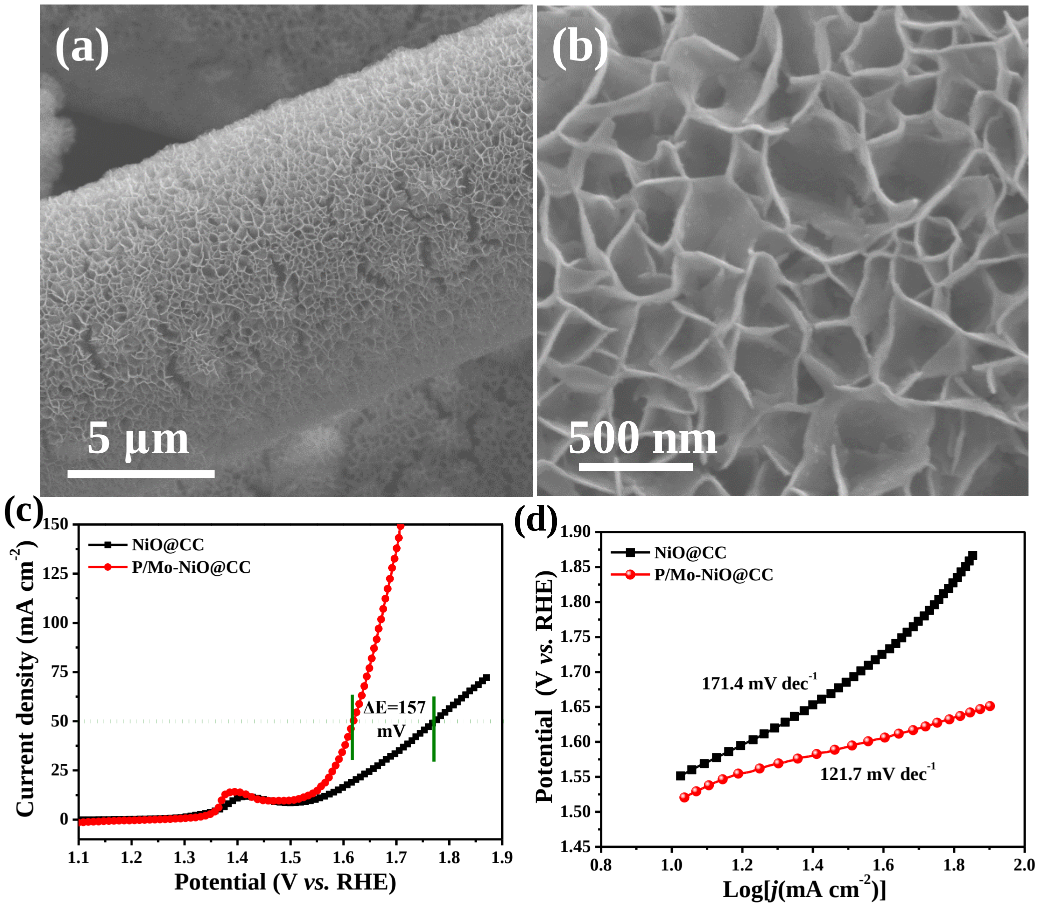

Figure S22. SEM images (a-b), LSV curves (c) and Tafel plots (d) of P/Mo-NiO@CC.

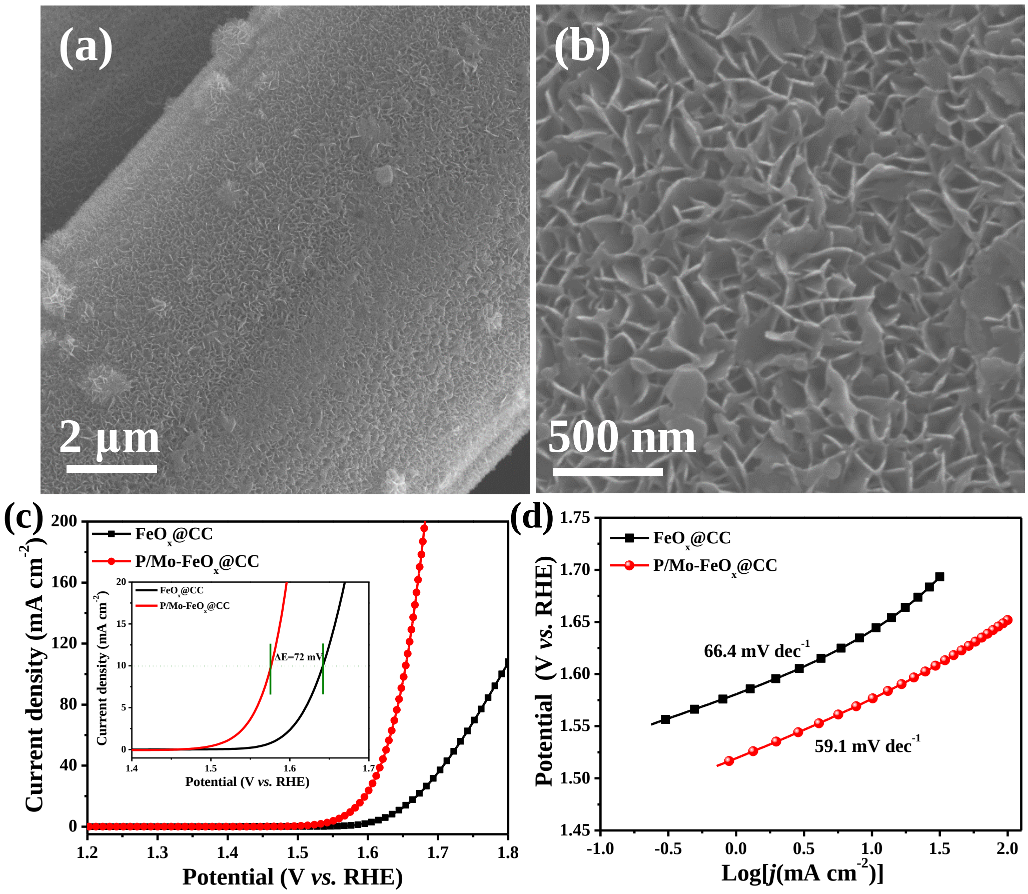

Figure S23. SEM images (a-b), LSV curves (c) and Tafel plots (d) of P/Mo-FeO_x@CC.

Table S1. The element contents by XPS analysis.

Element	Co_3O_4@CC	P/Mo- Co_3O_4@CC
Mo (at.%)	/	0.73
Co(at.%)	34. 22	19.37
O(at.%)	65.78	65.78
P(at.%)	/	14.12

Table S2. Quantitative analysis of XPS results of obtained samples.

Samples	Co^{2+}(%)	Co^{3+}(%)	area ratio of Co^{2+}/ Co^{3+}
Co_3O_4@CC	36.3%	63.7%	0.57
P/Mo-Co_3O_4@CC	66.5%	33.5%	1.99

Table S3. The element contents of the P/Mo-Co_3O_4@CC before and after stability test.

Element	XPS results		EDS results	
	before	after stability test	before	after stability test
Mo (at.%)	0.73	0.00[1]	3.75	3.43
Co(at.%)	19.37	26.69	14.18	24.75
O(at.%)	65.78	73.31	66.11	71.82
P(at.%)	14.12	0.00	15.96	0.00

[1] The content of this element is too low to be detected.

Table S4. Chemical composition of obtained materials by EDS results with different CV cycles.

Cycles	0	5	10	20	30	60	100
P (at.%)	15.96	8.14	1.23	0.97	0. 73	0.00	0.00

Table S5. The OER performance of the Co-based electrocatalysts.

Electrocatalysts	Electrolyte	Overpotential at j = 10 mA cm^{-2}	Tafel plot (mV dec^{-1}	Ref.
Co-PBA-plasma-2 h	1 M KOH	274 mV	53	[3]
$NiNO_{0+15}$	1 M KOH	300 mV	74	[4]
$Co_{1.8}Ni(OH)_{5.6}$@ $Co_{1.8}NiS_{0.4}(OH)_{4.8}$	0.1 M KOH	274 mV	45	[5]
NieCoP@C	1 M KOH	279 mV	54	[6]
Fe_1-$(Co_3O_4)_5$ H-NSs	1 M KOH	269 mV	53	[7]
PBA-5	1 M KOH	271 mV	53.7	[8]
Fe@BIF-91	1 M KOH	350	71	[9]
CeO_x/CoO_x	1 M NaOH	313 mV	66	[10]
$CoNi_{0.2}Fe_{0.05}$-Z-H-P	1 M KOH	329 mV	48.2	[11]
Co_3O_4C-NA	0.1 M KOH	290 mV	70	[12]
CoSe/$MoSe_2$	1M KOH	262 mV	54.9	[13]
5% Ni Promoted Mesoporous Co_3O_4	0.1 M KOH	381 mV	73	[14]
Co-UNMs	1 M KOH	307 mV	76	[15]
ER-Co_3O_4 NWs-2	1.0 M KOH	344 mV	50	[16]
Co_3O_4 nanosheets	0.1M KOH	300 mV	68	[17]
Co_3O_4-MTA	1M KOH	/	84	[18]
Co_3O_4-EC-0.50	1 M NaOH	377 mV	58.1	[19]
LCF-700	1 M KOH	293 mV	67	[20]
**P/Mo-Co_3O_4@CC**	**1 M KOH**	**265 mV**	**59.4**	**This work**

References

[1] X. Ren, F. Hou, F. Wang, X. Zhang, Q. Wang, *Int. J. Hydrogen Energy* **2018**, *43*, 22529-22537.

[2] X. Han, C. Yu, S. Zhou, C. Zhao, H. Huang, J. Yang, Z. Liu, J. Zhao, J. Qiu, *Adv. Energy Mater.* **2017**, *7*, 1602148.

[3] Y. Guo, T. Wang, J. Chen, J. Zheng, X. Li, K. K. Ostrikov, *Adv. Energy Mater.* **2018**, *8*, 1800085.

[4] J. Huang, Y. Sun, X. Du, Y. Zhang, C. Wu, C. Yan, Y. Yan, G. Zou, W. Wu, R. Lu, Y. Li, J. Xiong, *Adv. Mater.* **2018**, *30*, e1803367.

[5] B. Wang, C. Tang, H. F. Wang, X. Chen, R. Cao, Q. Zhang, *Adv. Mater.* **2019**, *31*, e1805658.

[6] X. Han, C. Yu, H. Huang, W. Guo, C. Zhao, H. Huang, S. Li, Z. Liu, X. Tan, Z. Gao, J. Yu, J. Qiu, *Nano Energy* **2019**, *62*, 136-143.

[7] Y. Li, F. M. Li, X. Y. Meng, X. R. Wu, S. N. Li, Y. Chen, *Nano Energy* **2018**, *54*, 238-250.

[8] W. Zhang, H. Song, Y. Cheng, C. Liu, C. Wang, M. A. N. Khan, H. Zhang, J. Liu, C. Yu, L. Wang, J. Li, *Adv. Sci.* **2019**, *6*, 1801901.

[9] T. Wen, Y. Zheng, J. Zhang, K. Davey, S. Z. Qiao, *Adv. Sci.* **2019**, *6*, 1801920.

[10] J. H. Kim, K. Shin, K. Kawashima, D. H. Youn, J. Lin, T. E. Hong, Y. Liu, B. R. Wygant, J. Wang, G. Henkelman, C. B. Mullins, *ACS Catal.* **2018**, *8*, 4257-4265.

[11] M. Wang, C. L. Dong, Y. C. Huang, Y. Li, S. Shen, *Small* **2018**, *14*, e1801756.

[12] T. Y. Ma, S. Dai, M. Jaroniec, S. Z. Qiao, *J. Am. Chem. Soc.* **2014**, *136*, 13925-13931.

[13] M. Yuan, S. Dipazir, M. Wang, Y. Sun, D. Gao, Y. Bai, M. Zhang, P. Lu, H. He, X. Zhu, S. Li, Z. Liu, Z. Luo, G. Zhang, *J. Mater. Chem. A* **2019**, *7*, 3317-3326.

[14] W. Song, Z. Ren, S. Y. Chen, Y. Meng, S. Biswas, P. Nandi, H. A. Elsen, P. X. Gao, S. L. Suib, *ACS Appl. Mater. Interfaces* **2016**, *8*, 20802-20813.

[15] X. Xiong, C. You, Z. Liu, A. M. Asiri, X. Sun, *ACS Sustainable Chem. Eng.* **2018**, *6*, 2883-2887.

[16] S. Liu, H. Cheng, K. Xu, H. Ding, J. Zhou, B. Liu, W. Chu, C. Wu, Y. Xie, *ACS Energy Lett.* **2019,** *4*, 423−429.

[17] L. Xu, Q. Jiang, Z. Xiao, X. Li, J. Huo, S. Wang, L. Dai, *Angew. Chem. Int. Ed.* **2016**, *55*, 5277-5281.

[18] Y. P. Zhu, T. Y. Ma, M. Jaroniec, S. Z. Qiao, *Angew. Chem. Int. Ed.* **2017**, *56*, 1324-1328.

[19] H. S. Jeon, M. S. Jee, H. Kim, S. J. Ahn, Y. J. Hwang, B. K. Min, *ACS Appl. Mater. Interfaces* **2015**, *7*, 24550-24555.

[20] S. Song, J. Zhou, X. Su, Y. Wang, J. Li, L. Zhang, G. Xiao, C. Guan, R. Liu, S. Chen, H. J. Lin, S. Zhang, J. Q. Wang, *Energy Environ. Sci.* **2018**, *11*, 2945-2953.
